# Penetrating aortic ulcer following endoscopic dilatation of esophageal anastomotic stenosis: a case report

**DOI:** 10.3389/fsurg.2025.1663479

**Published:** 2025-12-02

**Authors:** Jingjing Yang, Qinghua Wang, Wei Han

**Affiliations:** 1 Department of Gastroenterology, Kunshan First People's Hospital Affiliated to Jiangsu University, Kunshan, Jiangsu, China; 2 Department of Science and Education, Kunshan Women and Children’s Healthcare Hospital, Kunshan, Jiangsu, China; 3 Department of General Surgery, Kunshan Women and Children’s Healthcare Hospital, Kunshan, Jiangsu, China

**Keywords:** penetrating aortic ulcer, Ortner's syndrome, endoscopic balloon dilation, esophageal anastomotic stricture, endovascular repair, iatrogenic vascular injury, esophagectomy complications

## Abstract

**Background:**

Endoscopic balloon dilation serves as the first-line treatment for anastomotic strictures after esophagectomy. While generally safe, the procedure can, in rare instances, lead to severe vascular complications.

**Case presentation:**

A 64-year-old male with a prior history of esophagectomy for cancer presented with dysphagia due to an anastomotic stricture. Shortly after undergoing endoscopic balloon dilation, he developed hoarseness. Computed tomography (CT) identified a penetrating aortic ulcer (PAU) with contained rupture and an associated hematoma compressing the left recurrent laryngeal nerve, consistent with Ortner's syndrome. The patient was successfully managed with emergent thoracic endovascular aortic repair (TEVAR). At the three-month follow-up, the aortic stent remained, and his hoarseness had significantly improved.

**Conclusion:**

This rare case of a PAU and Ortner's syndrome following endoscopic dilation underscores the critical importance of vigilance for life-threatening vascular injuries after seemingly routine procedures. It highlights the necessity of considering such complications in the differential diagnosis of post-procedural symptoms and demonstrates the efficacy of endovascular repair as a definitive treatment option.

## 1 Introduction

Esophageal anastomotic stenosis is a common complication following esophageal cancer surgery, potentially leading to dysphagia and significantly impacting patients' quality of life. Endoscopic balloon dilation is widely regarded as an effective and safe treatment for esophageal stricture (1); however, common complications may include esophageal perforation, bleeding, and chest pain (2). Hoarseness can be caused by acute or chronic laryngitis, functional voice disorders, tumors, or neurogenic disorders; however, hoarseness resulting from endoscopic treatment is rare (3). A penetrating aortic ulcer, characterized by ulcerative lesions resulting from the rupture of atherosclerotic plaques in the aortic intima (4), represents a rare but serious complication after endoscopic therapy. Vigilance for potential complications following endoscopic procedures is crucial, as early detection and timely intervention are essential for improving patient outcomes. This study presents the case of a penetrating aortic ulcer following endoscopic balloon dilation, highlighting the importance of prompt recognition of such postoperative complications.

## 2 Case report

A 64-year-old male patient was admitted to Kunshan First People's Hospital in March 2023, reporting a six-month history of intermittent dysphagia.

### 2.1 Clinical timeline

The patient's clinical course is summarized as follows: He was initially diagnosed with an esophageal malignancy in March 2023. He underwent endoscopic submucosal dissection (ESD) on May 16, 2023, followed by a radical esophagectomy on June 10, 2023, for pathologically confirmed esophageal squamous cell carcinoma. Subsequently, an anastomotic stricture was identified. The critical sequence of events began with the first endoscopic balloon dilation on November 3, 2023 (designated as Day 0). A second dilation was performed on Day 3 (November 6), followed by the onset of hoarseness on Day 4 (November 7). Imaging on Day 5 (November 8) confirmed the diagnosis of a penetrating aortic ulcer, which led to thoracic endovascular aortic repair (TEVAR) on Day 11 (November 14).

His medical history included hypertension, hyperlipidemia, and diabetes mellitus, all managed for approximately one year. The patient was diagnosed with esophageal squamous cell carcinoma in March 2023, as detailed in the timeline above. An ESD was performed for a suspected local recurrence, and subsequent pathological analysis confirmed the diagnosis of locally recurrent esophageal squamous cell carcinoma. Esophagography revealed a postoperative anastomotic stricture without significant space-occupying lesions.

**Figure 1:**
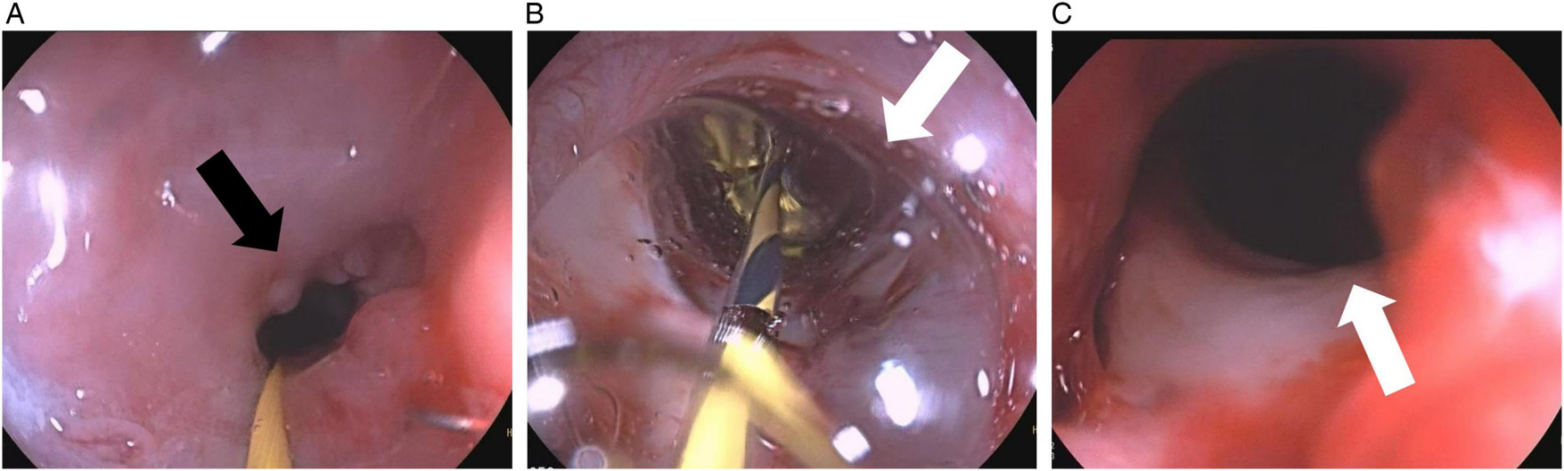
Endoscopic views of the esophageal anastomotic stricture during balloon dilation. **(A)** Pre-dilation view showing the severe anastomotic stricture (indicated by the black arrow). **(B)** Intra-operative view with the balloon dilator fully inflated across the stricture (indicated by the white arrow). **(C)** Post-dilation view demonstrating a widely patent lumen after successful dilation (indicated by the white arrow).

**Figure 2:**
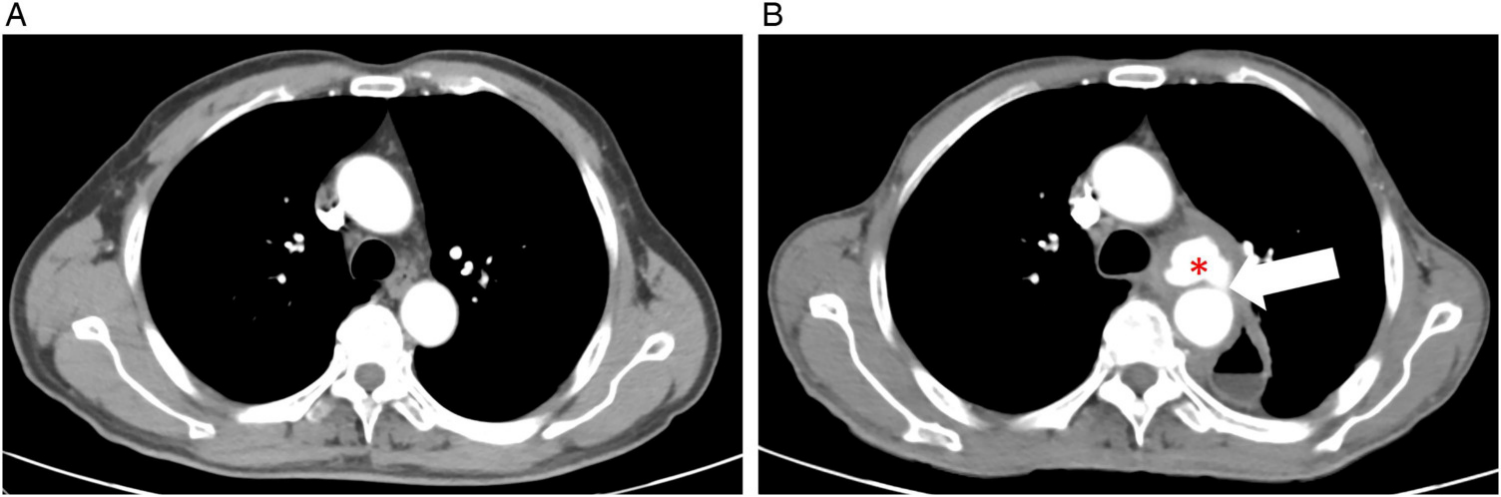
Contrast-enhanced computed tomography (CT) of the chest reveals the aortic injury. **(A)** Axial view from a pre-dilation contrast-enhanced CT (May 8, 2023), the radiological report of which described aortic wall calcification (indicating pre-existing atherosclerosis). **(B)** Axial view from a post-dilation contrast-enhanced CT (November 9, 2023) showing a penetrating aortic ulcer (PAU) with contained rupture (indicated by the white arrow), evidenced by a focal collection of contrast outside the lumen, and a surrounding hematoma (indicated by the asterisk).

**Figure 3:**
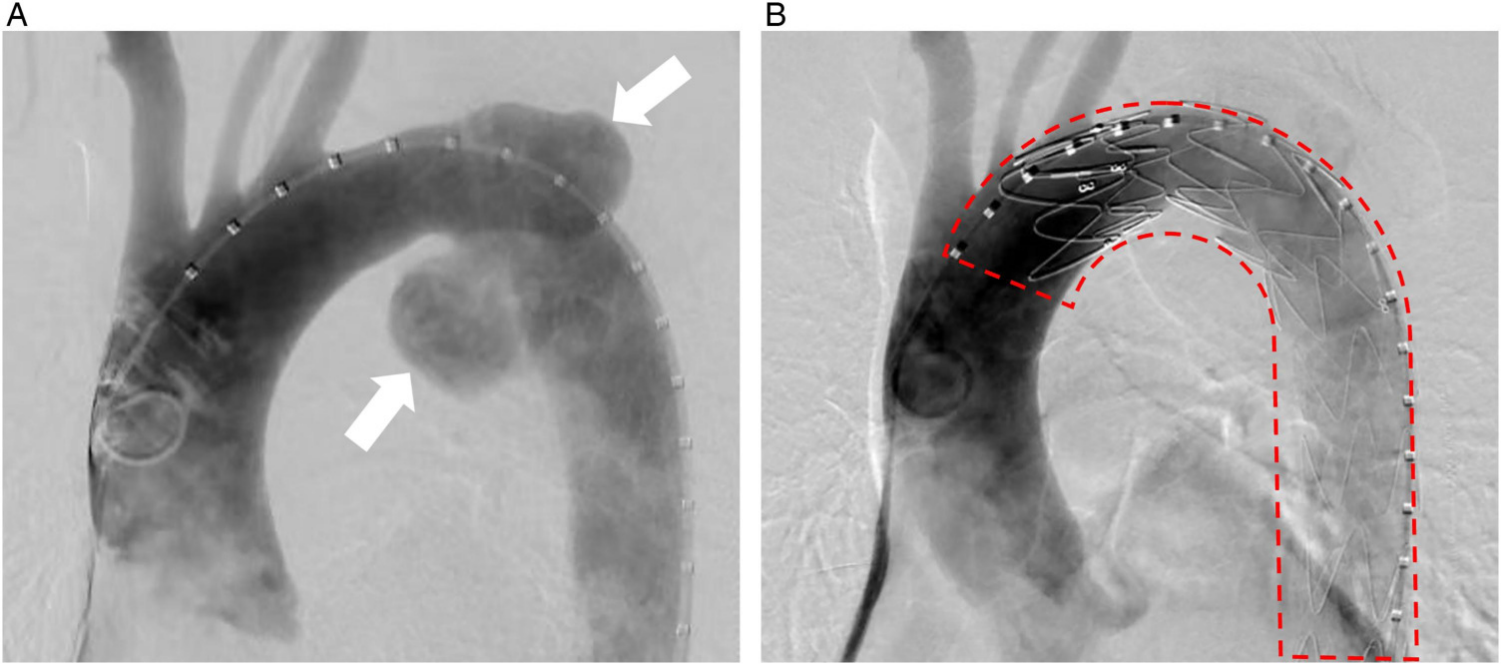
Angiographic confirmation and endovascular repair of the penetrating aortic ulcer. **(A)** Initial transfemoral aortography in the anteroposterior (AP) projection, revealing the penetrating aortic ulcer (indicated by the arrow) as a contrast-filled outpouching from the aortic wall. **(B)** Final aortography after thoracic endovascular aortic repair (TEVAR) showing successful exclusion of the ulcer by the deployed stent graft (outlined by the dashed line) with no evidence of contrast extravasation.

To address the stenosis, sequential endoscopic balloon dilations were performed. A second dilation was conducted three days after the initial procedure (Figure 1). Both procedures were targeted at the same anastomotic stricture level and were performed under endoscopic vision without fluoroscopic guidance. A cylindrical balloon catheter (Micro-Tech, Nanjing, China) was used in both dilations, inflated to a diameter of 12 mm at 6 atmospheres, with each inflation maintained for 3 min. On the following day, the patient reported new-onset throat discomfort and hoarseness. At the same time, initial suspicion included cricoarytenoid dislocation; direct laryngoscopy confirmed complete paralysis of the left vocal cord. Enhanced chest computed tomography (CT) subsequently revealed a penetrating aortic ulcer with contained rupture and associated hematoma formation (Figure 2). Given the patient's age and comorbidities, thoracic endovascular aortic repair (TEVAR) with a covered stent was successfully performed (Figure 3). At the three-month follow-up, the patient's hoarseness had completely resolved; however, his pharyngeal discomfort and dysphagia persisted due to the local progression of the recurrent esophageal malignancy, which required continued oncological management.

## 3 Discussion

The recurrent laryngeal nerve constitutes the primary motor nerve for the larynx and is vulnerable to compression or injury due to a variety of cervicothoracic disorders, including aortic aneurysms, mediastinal tumors, surgical interventions, trauma, exposure to neurotoxins, and viral infections (5). Such damage may produce vocal-cord paralysis and hoarseness, a symptom often attributed to benign factors. When hoarseness persists or occurs suddenly without an obvious cause, it may indicate serious underlying pathology and warrants further evaluation. Ortner syndrome, also known as cardiac-vocal cord syndrome, represents a rare etiology of hoarseness characterized by vocal cord paralysis stemming from cardiovascular conditions (6). Specifically, aortic aneurysms, pulmonary hypertension, and other primary cardiovascular diseases that induce dilation of the aorta or pulmonary artery, along with left atrial enlargement, can induce hoarseness by exerting pressure on the recurrent laryngeal nerve, located between the aortic arch and the pulmonary artery (7). Direct laryngoscopy frequently reveals reduced activity or paralysis of the left vocal cord in affected patients. In this case, the patient's postoperative discomfort is attributed to compression of the left recurrent laryngeal nerve by penetrating aortic ulcers and an intramural aortic hematoma.

A penetrating aortic ulcer is defined as the ulceration of an atherosclerotic plaque within the aorta that breaches the internal elastic lamina and extends into the media, typically associated with varying degrees of intramural hematoma formation (8). PAU represents a complex and potentially life-threatening condition characterized by rapid progression (9), which can culminate in catastrophic aortic rupture or other severe complications. The true prevalence of PAU remains elusive; however, risk factors such as smoking, advanced age, hypertension, hypercholesterolemia, and other associated comorbidities may increase the likelihood of developing this condition (10). On computed tomography (CT) imaging, PAUs characteristically present as contrast-filled, pouch-like protrusions within the thickened aortic wall, often accompanied by adjacent intramural hematoma formation (11). Given that PAUs are frequently identified as localized segmental wall lesions, endovascular stent grafting is considered the primary therapeutic modality, particularly for elderly patients with multiple underlying comorbidities and a prohibitively elevated risk associated with open surgery (12). Our management strategy with thoracic endovascular aortic repair (TEVAR) is strongly supported by contemporary clinical practice guidelines, which recommend endovascular repair as the first-line therapy for contained ruptures and traumatic injuries of the descending thoracic aorta (13–15).

The development of a PAU following endoscopic dilation likely results from a combination of patient-specific vulnerability and procedural factors. In our patient, the pre-existing aortic calcification seen on CT signifies a stiff, atherosclerotic aortic wall with limited elasticity. We postulate that the mechanical force exerted by the balloon dilation directly traumatized this compromised aortic segment. This trauma likely caused focal disruption of the atherosclerotic plaque and underlying intima, initiating PAU and intramural hematoma formation. While underlying atherosclerosis creates a predisposed environment, the clear temporal relationship between the procedure and the onset of symptoms strongly supports an iatrogenic etiology rather than a spontaneous event. While our case of a penetrating aortic ulcer following balloon dilation is exceptionally rare, it exists within a spectrum of documented, though scarce, iatrogenic aortic injuries resulting from esophageal interventions. The literature contains instructive parallels that underscore the potential for catastrophic vascular complications. Tezcan et al. reported a direct iatrogenic aortic injury in a child during esophagoscopy for foreign body removal, where a balloon dilator was subsequently used in an attempt to control the bleeding, necessitating emergent open surgical repair (16). This case highlights that mechanical instrumentation near the esophagus, even in the absence of pre-existing aortic disease, can directly traumatize the aortic wall. Furthermore, the risk extends beyond dilation to other procedures. Liao et al. described a fatal aortoesophageal fistula (AEF) that developed secondary to a pseudoaneurysm caused by an esophageal stent placed for malignancy (17). This illustrates that sustained pressure from an intraluminal device against the aorta, especially in the context of a compromised tissue plane, can lead to erosion and fistulization. Our case contributes a critical nuance to this clinical spectrum: unlike the immediate, massive bleeding seen in direct aortic injury or the classic “herald bleed” of an AEF, our patient presented with a *contained* aortic injury (PAU) whose primary manifestation was a neurologic symptom (Ortner's syndrome). This distinctive presentation underscores the need to consider vascular etiologies even in the absence of overt hemorrhagic signs, particularly when hoarseness arises post-procedure. The collective evidence from these cases solidifies the existence of this catastrophic complication pathway and mandates a high index of suspicion during any esophageal intervention close to the aorta.

### 3.1 Preventive strategies and clinical implications

The grave nature of these complications mandates a proactive approach to prevention. Based on our experience and the literature, we propose several strategic considerations for high-risk patients (e.g., those with known atherosclerosis, severe aortic calcification on prior imaging, or history of thoracic surgery/radiotherapy). First, a careful pre-procedural evaluation with contrast-enhanced CT should be considered to assess the anatomical relationship between the esophagus, the stricture, and the adjacent aorta. Second, during dilation, the principle of “less is more” should be adopted; using the lowest effective balloon inflation pressure and avoiding over-dilation may reduce the risk of mechanical trauma. Finally, establishing a high index of suspicion and a low threshold for post-procedural monitoring is crucial. Patients and healthcare staff should be educated to report any warning symptoms, such as new-onset hoarseness, chest pain, or odynophagia, immediately. These symptoms should prompt rapid cross-sectional imaging to rule out a vascular injury.

## 4 Conclusions

In conclusion, the case delineated in this study, which involves aortic penetrating ulcers and hematomas following endoscopic balloon dilation, resulting in left vocal cord paralysis, represents a clinically rare but life-threatening phenomenon. This report serves as a critical reminder of the potential for serious vascular injury during seemingly routine endoscopic procedures. It underscores the importance of vigilant patient selection, consideration of pre-procedural imaging in high-risk cases, and the need for heightened awareness among endoscopists to promptly identify and manage such catastrophic complications.

## Data Availability

The original contributions presented in the study are included in the article/Supplementary Material, further inquiries can be directed to the corresponding authors.
